# Pain differences in cognitive impairment appear only during active motor tasks

**DOI:** 10.3389/fpain.2026.1849507

**Published:** 2026-06-22

**Authors:** Stefan Lautenbacher, Vivien Schreiber, Clemens Grupp, Claus-Christian Carbon, Heiko Kellner, Miriam Kunz

**Affiliations:** 1 Department of Medical Psychology and Sociology, Medical Faculty, University of Augsburg, Augsburg, Germany; 2 Department of Clinical Biopsychology, Otto-Friedrich University, Bamberg, Germany; 3 Medizinische Klinik III mit Zentrum für Altersmedizin, Klinikum der Sozialstiftung Bamberg, Bamberg, Germany; 4 Department of General Psychology and Methodology, Otto-Friedrich University, Bamberg, Germany

**Keywords:** dementia, experimental pain, impaired cognition, living lab, movement-related pain, movement planning

## Abstract

Cognitive impairment in older adults has been associated with altered pain processing and potentially increased pain vulnerability, although findings are inconsistent. We hypothesized that cognitive impairment has a stronger impact on movement-related pain in everyday contexts than on pain sensitivity assessed in passive laboratory settings. Thirty-eight cognitively healthy and 38 cognitively impaired older adults (50% women; groups age- and sex-matched), classified using the CERAD scale (Consortium to Establish a Registry for Alzheimer's Disease), completed standardized motor tasks (walking, bed mobility, lifting tasks) and experimental pressure and heat pain assessments. Pain intensity (NRS) was recorded for all tasks. Perceived exertion was assessed during motor tasks, and clinical pain status was determined via a structured interview. Groups did not differ in baseline clinical pain or experimental pain sensitivity. However, cognitively impaired participants reported significantly higher movement-related pain during motor tasks. In a blockwise regression model, movement-related pain was significantly predicted by clinical pain and experimental pain sensitivity; however cognitive status (CERAD) explained additional variance to a similar amount. In conclusion, reduced cognitive functioning significantly predicted increased pain during active, everyday motor tasks. This may reflect diminished capacities for recall, planning, and execution of pain-minimizing movement strategies. These findings underscore the importance of assessing pain in behaviorally relevant contexts where movement provokes pain, to better understand pain mechanisms in cognitively impaired older adults.

## 1 Introduction

Pain in older adults is influenced not only by structural pathology of somatic tissues and neural pathways but also by individual pain vulnerability (1, 2). A central component of pain vulnerability is pain sensitivity (3), which is typically assessed under controlled laboratory conditions using standardized noxious stimuli while participants remain passive. Such paradigms allow precise regulation of stimulus intensity, duration, and timing and are considered the gold standard for quantifying nociceptive processing. Commonly used outcome measures include pain thresholds and ratings of sensory pain intensity (4, 40).

In everyday life, however, individuals rarely remain passive when exposed to potentially painful stimuli. Instead, they actively anticipate, modulate, and respond to noxious input through behaviors that may mitigate or exacerbate pain (6). These pain-related actions can be systematically examined in living-lab settings, which maintain experimental control while enabling ecologically valid, naturalistic behavior (7).

Cognitive capacities are likely to play a crucial role in shaping these pain-related actions (8). Memory enables individuals to draw on previous pain experiences and apply effective coping strategies, while motor planning supports the anticipation and execution of protective movements that minimize mechanical strain or noxious stimulation (9). Both memory and motor planning functions are frequently compromised in individuals with cognitive impairment and have the potential to modulate pain experiences during everyday activities. These functions rely on distributed prefrontal, medial temporal, and fronto-striatal networks, including the dorsolateral prefrontal cortex, hippocampus, and basal ganglia, rather than primarily supporting nociceptive processing. Importantly, these regions are among the structures affected early in different types of dementia, including Alzheimer's disease and frontotemporal dementia (10, 11).

Based on these conciderations, we investigated older adults with and without cognitive impairment in a living-lab paradigm involving potentially painful everyday motor tasks (e.g., walking, bed mobility, lifting). We hypothesized that the pain experienced during these motor tasks is influenced not only by participants' clinical pain and experimental pain sensitivity, but also by their cognitive status. To test this hypothesis, we conducted group comparisons between cognitively healthy and cognitively impaired older adults and performed regression analyses examining clinical pain, experimental pain sensitivity as well as cognitive functioning as predictors of movement-related pain intensity.

## 2 Methods

### 2.1 Participants

Our sample is comprised of 38 cognitively healthy older individuals as well as 38 cognitively impaired older individuals (≥65 years). Participants were recruited via advertisements and flyers by inviting individuals to participate in memory testing, and in collaboration with the Sozialstiftung Bamberg hospital. Participants were excluded if they had a history of a major neurological or psychological disorder other than dementia and participants did not take analgesic medication, alcohol or CNS-active drugs on the day of testing.

Group allocation was determined based on performance on the CERAD (Consortium to Establish a Registry for Alzheimer's Disease; Morris, 1989) neuropsychological battery, a widely used international assessment tool for cognitive functioning. The CERAD battery comprises several subtests, including a semantic fluency task, a picture naming task, a word-list learning task with delayed recall and recognition, and a visual construction task, each designed to assess different cognitive domains. To provide a single measure of overall cognitive performance, Chandler et al. (12) proposed a total CERAD score by summing the raw scores of the subtests. This total score has been shown to have good psychometric properties, including reliability and validity; also, in German populations (13). Following the recommendation by Chandler et al. (12), a cut-off score of 77 was used to classify participants as either cognitively healthy or cognitively impaired (possibly early-stage dementia), providing a standardized criterion for group assignment. After this group classification, participants in the cognitively healthy and cognitively impaired groups (from an initial total sample of 108 recruited participants), were then matched by age and sex using R (pairwise matching), yielding 38 participants per group and a total sample of 76 participants. *Post hoc* power analyses performed with GPower (14) indicated a power of 0.70 (group comparisons) and 0.95 (regression model) for our sample size, suggesting that the study had a sufficient probability of detecting true effects.

Table 1 summarizes demographic characteristics, total CERAD scores, and clinical pain baseline measures according to interview. The groups were comparable with respect to clinical pain, with 76% of participants in each group reporting pain in the preceding seven days and demonstrating similar pain intensity levels. In addition to the CERAD total score, participants completed the Mini-Mental State Examination [MMSE; (15)]. The cognitively impaired group (mean = 27.4, SD = 2.2) scored significantly lower than the cognitively healthy group (mean = 28.4, SD = 1.6) (*p* = .012), indicating impairment of possible early-stage or mild dementia in the cognitively impaired group.

**Table 1:** Sample characteristics stratified by cognitive status of the participants.

	Cognitively healthy	Cognitively impaired	*p* (group difference)
*N*	38	38	
male/female	19/ 19	19/19	n.a.
Age (mean, SD)	80.2 (5.4)	80.1 (5.3)	.475
MMSE (mean, SD)	28.4 (1.6)	27.4 (2.2)	.012*
CERAD (total score)^a^	85.0 (5.0)	69.1 (7.3)	<.001***
Clinical pain			
Painful sites (number)	2.9 (2.0)	3.5 (2.9)	.304
Pain in the last 7 days: no/yes	10/28	10/28	n.a.
Pain in the last 7 days: intensity (mean, SD; 1–5)	2.7 (1.3)	2.9 (1.2)	.658

^a^ The cut-off score distinguishing cognitively healthy from cognitively impaired individuals (dementia) is 77.0 [based on (12)].

*p < 0.05, ***p < .001.

The study was conducted in accordance with the Declaration of Helsinki and approved by the ethics committee of the University of Bamberg (2021-04/18). All participants still had legal capacity. After being informed about the study in a way adjusted to the individual intellectual capacities, a written informed consent was obtained from all participants. We monitored the patients continuously for any signs of disproportionate discomfort (verbally or non-verbally), in which case we stopped testing immediately. Participants received a minor monetary compensation for their participation.

### 2.2 Procedures and materials

The study comprised two testing sessions. During Session 1 (approximately 1 h), demographic information, cognitive functioning (CERAD), and clinical pain were assessed using a structured geriatric pain interview [GPI, (16)]. Session 2 was the pain testing session and took place 2–14 days later and lasted approximately 1.5 h. During this session, experimental pain sensitivity was measured using applied pressure and heat stimuli, and participants performed everyday whole-body motor tasks, such as lifting a box and walking. Participants provided self-report of pain intensity and exertion during session (physiological responses were also recorded but are not included in the present article.) All testing took place in a living-lab environment with controlled lighting (simulated daylight, 5,500 K) and temperature (21 °C).

#### 2.2.1 Session 1

##### 2.2.1.1 Cognitive functioning

The Consortium to Establish a Registry for Alzheimer's Disease (CERAD) provided a neuropsychological battery (17, 41) assessing multiple cognitive domains, including episodic memory (e.g., word list learning and delayed recall), language, visuoconstructional abilities (constructional praxis), and executive functioning (e.g., verbal fluency), across seven standardized subtests. Administration typically takes 30–45 min. The battery is widely used in both clinical and research settings for the assessment of cognitive decline and has demonstrated good reliability and validity, particularly for the detection of Alzheimer's disease and mild cognitive impairment (18). In this study, a composite CERAD total score was used as an index of global cognitive performance, with higher scores indicating better cognitive functioning. As described above, a cut-off score of 77 was applied to classify participants as either cognitively healthy or cognitively impaired, following the recommendation by Chandler et al. (12).

##### 2.2.1.2 Clinical pain at baseline

The Geriatric Pain Interview [Schmerzinterview für geriatrische Patienten; (16)] is a structured instrument designed to assess clinical pain in elderly populations. It evaluates multiple dimensions of pain, including location, intensity, frequency and duration, using both categorical and numerical scales. Administration typically takes 10–20 min. The interview has been incorporated into expert recommendations and German geriatric pain guidelines, including those addressing pain assessment in patients with mild to moderate cognitive impairment (19). For the present analyses, we focus particularly on the clinical pain intensity item (Item 5).

#### 2.2.2 Session 2

##### 2.2.2.1 Experimental heat and pressure stimuli (pain sensitivity)

When assessing pain sensitivity, we followed common experimental pain protocols (20, 21) and applied pressure as well as heat stimuli.

*Pressure pain:* Pressure stimuli were administered using a pressure algometer (Algometer Type II, SOMEDIC Electronics, Hörby, Sweden) with a probe area of 1 cm^2^, following the protocol described by Bunk et al. (20). Four pressure intensities (50, 200, 400, and 500 kPa) were applied in ascending order to the left and right shoulder (above the trapezius muscle, midway between the neck and shoulder line) and to the left and right inner forearm (midway between the wrist and elbow flexion). This resulted in a total of 16 pressure stimuli (4 intensities × 2 body sides × 2 stimulation sites). At each stimulation site, pressure was steadily increased over 2 s until the target intensity was reached and then maintained for 5 s. The decision for only applying ascending series of stimuli was primarily based on ethical considerations when working with cognitively impaired individuals, namely to (i) minimize anxiety and (ii) allow immediate termination of the procedure if participants showed signs of severe distress.

*Heat pain:* Similarly, as for pressure stimulation we chose a procedure resembling everyday experiences by using hot water immersion rather than technically more demanding thermode stimulation. Heat pain sensitivity was assessed by asking participants to immerse their right hand, approximately 2 cm above the wrist, into a water-filled sink maintained at a constant temperature of 46.5 °C using a thermostatically controlled sous-vide cooker (SV 100 Professional, Steba), following Lautenbacher et al. (21). Each immersion lasted 3 s and was repeated three times, followed by a 10-second pause. This sequence was repeated three times, resulting in a total of nine immersions (3–3–3). Because immersion depth and duration could not be perfectly standardized, we refrained from introducing further intensity variations in the protocol.

##### 2.2.2.2 Everyday motor challenges

**Figure 1:**
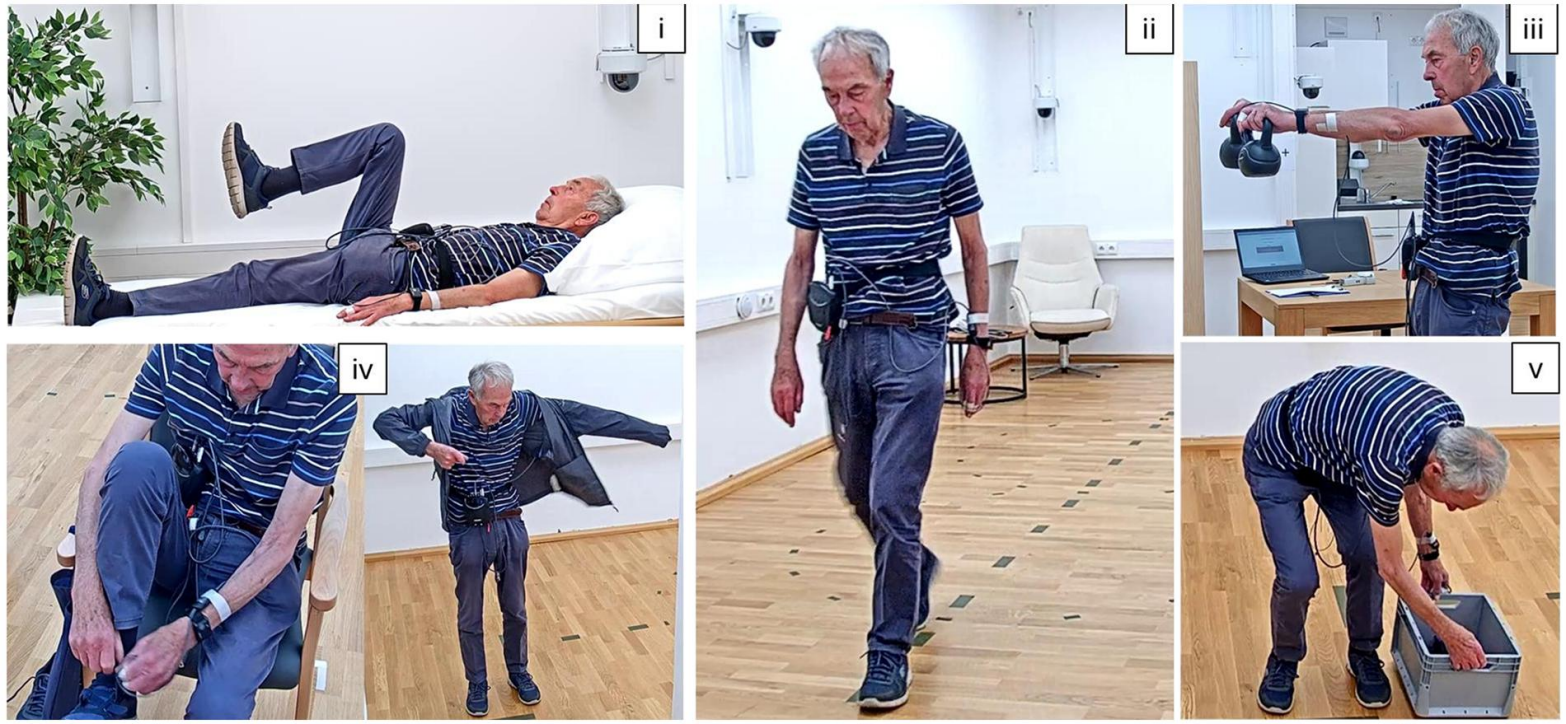
A paricipant performing the various everyday motor tasks. **(i)** Structured bed-based movements, **(ii)** walking, **(iii)** lifting kettlebell, **(iv)** putting on shoes and jacket, **(v)** lifting box. The participant provided informed consent for the use of their image in this figure.

We asked participants to perform a variety of everyday motor tasks that have the potential to elicit pain in older individuals (see photos depicting all challenges in Figure 1).

*Structured bed-based movements:* Following the MOBID-2 Pain Scale protocol (22), participants were guided through a series of standardized bed-based movements to elicit pain-related risk behaviors. These movements included opening and closing the hands, lifting each arm individually, flexing and extending the ankles and knees, lifting the legs (see Figure 1i), turning to both sides, sitting up in bed, and standing from the bed.

*Walking:* Participants were instructed to walk six-meter straight line twice (see Figure 1ii), followed by six repetitions of a figure-eight course marked on the floor, alternating clockwise and counterclockwise, as described by Belluscio et al. (23).

*Lifting kettlebell:* Participants stood in front of a shelf and lifted two 2-kg kettlebells from the middle board (86 cm) to shoulder height (see Figure 1iii) and the top edge of the shelf (160 cm), holding each position for 5 s before returning the weights to the middle board [adapted from Thibault et al. (24)]. They then lifted the kettlebells from the bottom board (9 cm) to the middle board following a 5-second pause. Each sequence was performed twice, once at a closer distance (28 cm) and once at a farther distance (60 cm) from the shelf. This task was designed to simulate everyday activities, such as handling or reorganizing objects on a kitchen shelf.

*Putting on shoes and jacket:* Participants were provided with shoes and a jacket to perform everyday dressing tasks (see Figure 1iv). While seated in a height-adjusted chair with feet flat on the floor, they put on and removed the provided shoes sequentially, starting with the right foot, using both hands to pull the foot toward them without crossing their legs. For the jacket, participants stood upright, inserted their dominant arm followed by the other arm, and closed the zipper.

*Lifting box:* Following (25), participants lifted a weighted box (40 cm × 30 cm × 22 cm; maximum 4 kg) from the floor on one side, held it upright for 1 s, and placed it on the opposite side, alternating sides (see Figure 1v). Each participant performed a total of 10 trunk rotation movements. This task was designed to simulate everyday activities such as lifting and moving a crate of drinks.

#### 2.2.3 Outcome variables

*Self-report of pain:* After each experimental stimulus and upon completing each motor challenge, participants were asked to indicate the maximum pain they experienced using an 11-point Numerical Rating Scale (NRS), with 0 representing “no pain” and 10 representing “worst pain imaginable.” For subsequent analyses, pain ratings were averaged separately across (i) all experimental heat and pressure stimuli to quantify experimental pain sensitivity, and (ii) all motor challenges to quantify pain elicited by everyday motor tasks.

*Self-report of physical exertion:* Participants also rated their maximum physical exertion during each motor task using an 11-point Numerical Rating Scale (0 = no exertion, 10 = maximal exertion) to control for potential differences in exertion that could influence pain responses. Exertion was not assessed following pressure or heat stimuli, as these tasks did not involve significant motor activity. Again, ratings were averaged across all motor challenges.

### 2.3 Statistical analysis

Group differences between cognitively healthy and cognitively impaired participants in pain ratings of the experimental stimuli and following everyday motor challenges were analyzed using one-tailed independent-samples *t*-tests, based on the *a priori* hypothesis that cognitively impaired participants would report higher pain levels. Although evidence on the impact of cognitive impairment on pain is mixed, there is more evidence for increased pain responses in cognitively impaired individuals compared with cognitively unimpaired individuals [for reviews see (26, 27)]; thus, supporting our directional hypothesis. In contrast, exertion ratings were compared between groups using two-tailed independent-samples *t*-tests, as no directional hypothesis was specified.

Depending on the results of the group comparisons additional analyses were conducted to examine in more detail the contribution of cognitive status to pain experienced during motor tasks. Specifically, blockwise regression analyses were performed to determine the extent to which cognitive status (CERAD) explained additional variance in movement-related pain beyond that accounted for by clinical pain at baseline (GPI) and pain sensitivity (pain ratings of experimental stimuli). This approach allowed for a direct comparison of the incremental variance explained by cognitive status relative to the two established pain-related predictors.

All statistical analyses were conducted using SPSS (Version 31). Statistical significance was set at *α* < .05. Effect sizes are reported as Cohen's *d*.

## 3 Results

### 3.1 Group differences in pain sensitivity (ratings of the experimental heat and pressure stimuli)

**Figure 2:**
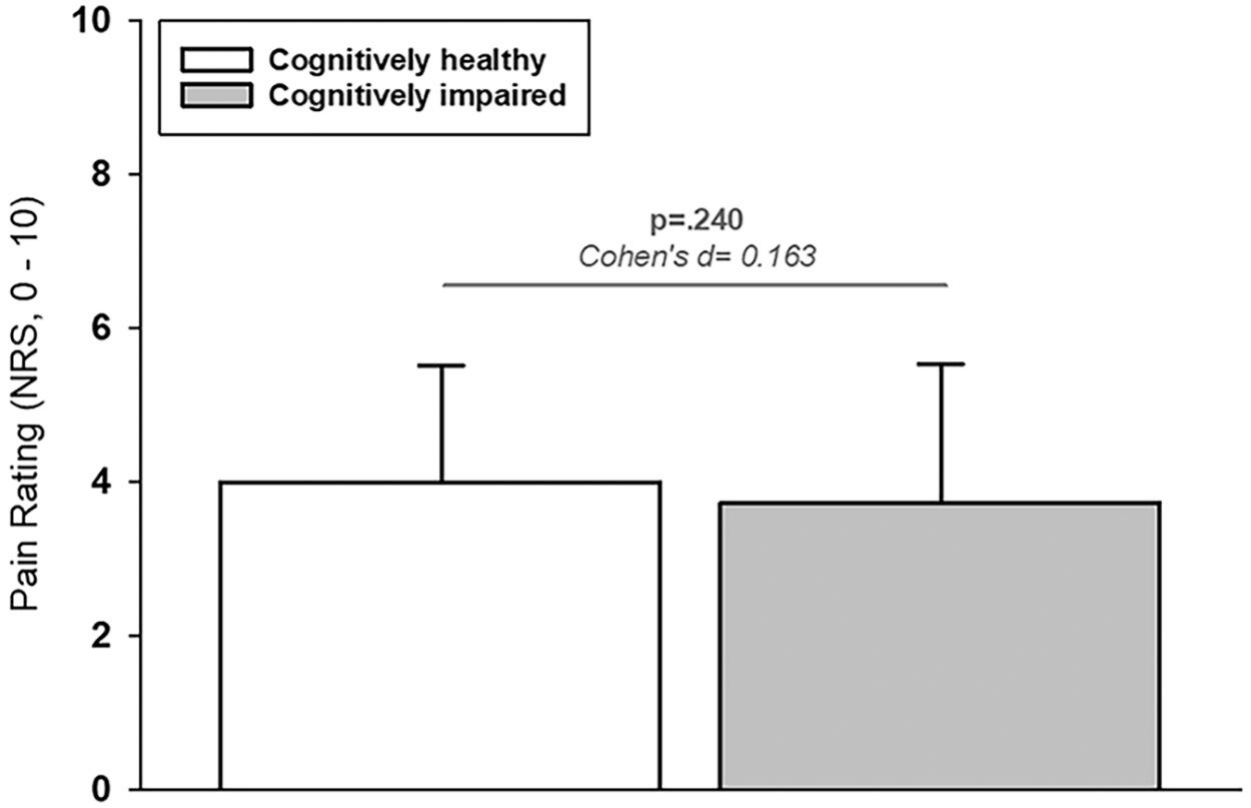
Experimental pain sensitivity. Mean and standard deviation of the pain ratings averaged over the two experimental pain tests (heat, pressure); *p*-values of the one-sided *t*-test and Cohen's *d* are given.

We found no significant differences in experimental pain sensitivity between cognitively healthy and cognitively impaired individuals [T(74) = 0.71; *p* = .240]. As can be seen in Figure 2, both groups rated the experimental heat and pressure stimuli as comparably painful.

### 3.2 Group differences in pain ratings of the everyday motor challenges

**Figure 3:**
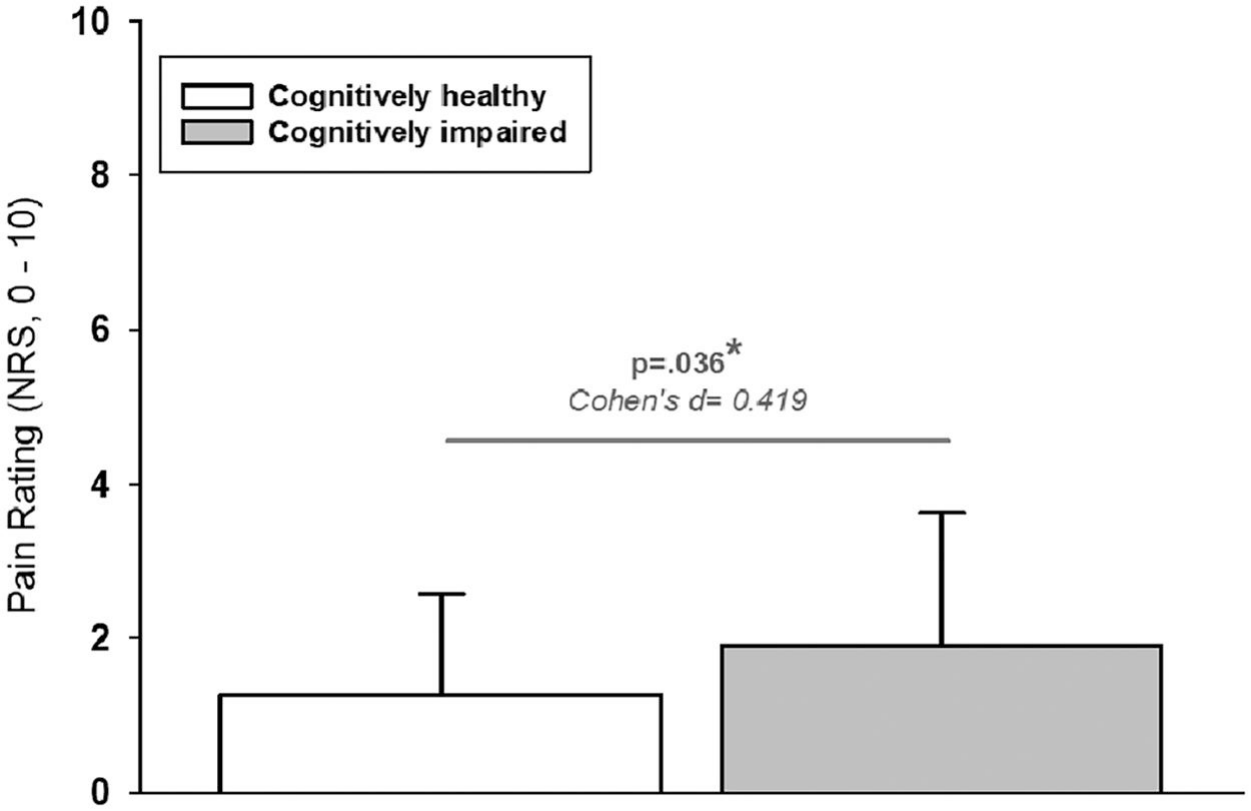
Evserday motor challenges—pian ratings. Mean and standard deviation of the pain ratings averaged across the five tasks (see Figure 1); *p*-values of the one-sided *t*-test and Cohen's *d* are given.

We found significant differences in pain ratings of the everyday motor tasks between cognitively healthy and cognitively impaired individuals [T(74) = –1.83, *p* = .036]. As shown in Figure 3, the cognitively impaired group gave higher pain ratings during the motor challenges. Cohen's *d* indicated a moderate effect size. Follow-up comparisons of pain ratings for each motor challenge using Mann–Whitney U tests revealed that cognitively impaired participants particularly rated structured bed-based movements (*p* = .015) and walking (*p* = .004) as more painful than cognitively healthy participants; all other comparisons did not reach significance (*p* > .05).

### 3.3 Group differences in exertion ratings of the everyday motor challenges

**Figure 4:**
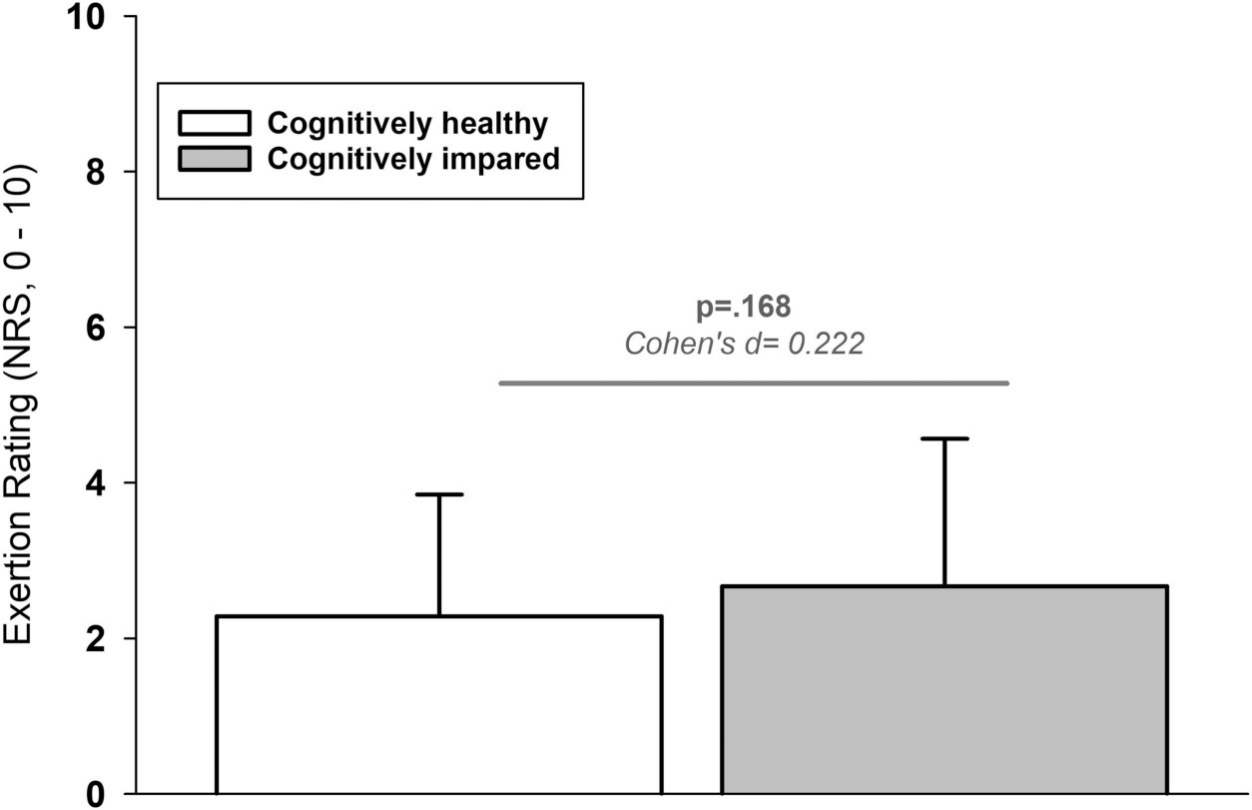
Evserday motor challenges—exertion ratings. Mean and standard deviation of the exertion ratings across the five motor tasks (see Figure 1); *p*-values of the two-sided *t*-test and Cohen's *d* are given.

We found no significant differences in exertion ratings between cognitively healthy and cognitively impaired individuals [T(74)=−0.97; *p* = .337]. As can be seen in Figure 4, both groups reported comparable levels of exertion during the motor challenges.

### 3.4 Regression analysis to investigate what role cognitive status plays to explain variance in pain responses to everyday motor challenges

Given that significant group differences were observed only in pain responses to everyday motor challenges, we further investigated factors contributing to variations in pain responses to the motor challenges. It seems likely that pain responses during motor challenges might be influenced by the individual's clinical pain at baseline as well as by his/her general pain sensitivity assessed via experimental stimuli. More importantly for the present study was the question to what extent does the cognitive functioning explain additional variance in pain responses to everyday motor challenges beyond that of pain-variables. To address this, we conducted a blockwise regression analysis across the whole sample (outcome variable “pain responses to everyday motor tasks”). In the first block, clinical pain at baseline, assessed via the GPI [item, 5 (16), see Table 1], was entered as predictor. In the second block, experimental pain sensitivity (experimental heat and pressure pain ratings) was included as predictor. In the final step, the CERAD total score was added to examine the unique contribution of cognitive functioning to the variance in pain responses to everyday motor challenges.

**Figure 5:**
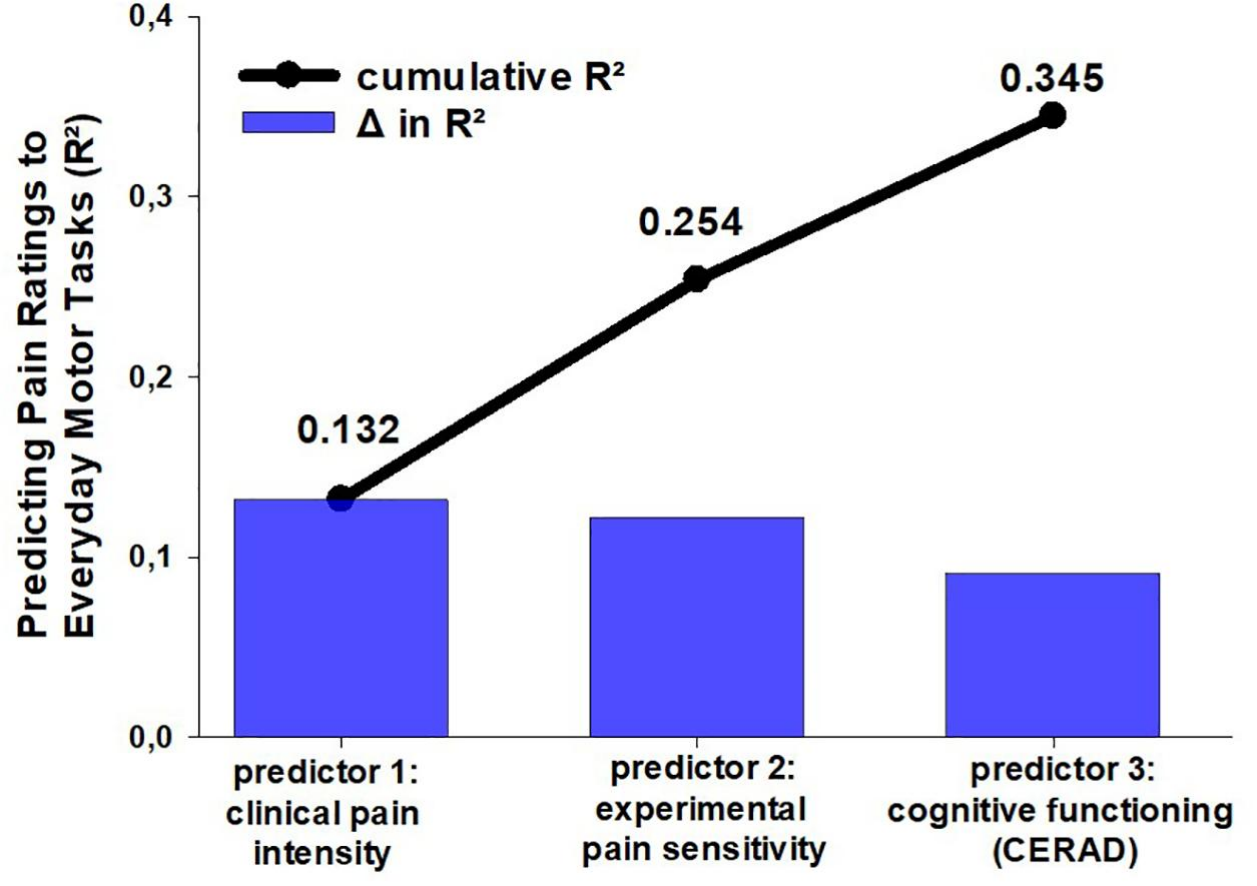
Predicting pain during active, everyday motor challenges. Linear regression analysis (blockwise) showing the predictive power of (1) “clinical pain” (2) “experimental pain sensitivity” and (3) “cognitive functioning” to explain variance in pain responses to everyday motor challenges.

We found that considering clinical pain at baseline (13.2% explained variance) and experimental pain sensitivity (additional 12.2%), i.e., the pain variables, significantly explained 25% of variance of the pain ratings in the motor tasks (see Table 2 and Figure 5). Despite of the considerable level of variance already explained, inclusion of cognitive functioning (CERAD total score) in a next block significantly accounted for an additional 9.1% of variance [Δ*R*^2^ = 0.091, *R*^2^ = 0.345, adjusted *R*^2^ = 0.318, *F* change (1,72) = 10.03, *p* = .002]; resulting in an impressive 35% of explained variance for the whole model (see Figure 5 and Table 2). These results indicate that, beyond clinical pain and experimental pain sensitivity, cognitive functioning contributes significantly to explaining individual differences in pain responses to everyday motor challenges.

**Table 2:** Blockwise linear regression with (1) clinical pain, (2) experimental pain sensitivity, and (3) cognitive functioning predicting pain responses during everyday motor tasks.

Step	Predictor	*β*	*t*	Total *r*^2^	Δ*r*^2^	*p* (Δ*r*^2^)
1	Clinical pain intensity (GPI, item 5)	.227	2.22	.132	.132	.001
2	Experimental pain sensitivity (pressure/heat)	.402	3.92	.254	.122	<.001
3	Cognitive functioning (CERAD)	−0.303	−3.17	.345	.091	.002

## 4 Discussion

The present study investigated whether individuals with cognitive impairments differ from cognitively healthy participants in pain outcomes during everyday motor tasks, and whether pain at baseline, pain sensitivity and cognitive status contribute to these outcomes. Our findings indicate that cognitively impaired individuals rated everyday motor tasks as more painful than cognitively healthy individuals, despite reporting similar levels of exertion. In contrast, the groups did not differ in experimentally assessed pain sensitivity to heat and pressure stimuli, nor in clinical pain at baseline.

The findings on pain sensitivity are consistent with previous research showing that individuals with mild to moderate dementia do not reliably differ from cognitively healthy individuals in ratings of experimentally induced pain (20, 28, 29). The present results suggest a complementary perspective: group differences in pain ratings emerged only in active conditions involving motor tasks, whereas no differences were observed in the passive condition with externally applied stimuli, as typically used in experimental pain research. However, we need to mention that there appears to be contradictory evidence to our findings: Horgas et al. (30) also used active movement tasks (sitting, standing, and walking) but reported lower pain ratings in cognitively impaired individuals. Their memory load, however, was substantially higher, as they collected a single rating after a 10-min series of several motor tasks, whereas we obtained ratings immediately after each individual motor task. Therefore, the results are difficult to compare.

A key distinction between the active and passive condition in our study is that, in the active condition, participants generated the noxious event through their own movements, making the pain somewhat self-generated, whereas in the passive condition the noxious stimulus was externally applied according to a standardized protocol. It is plausible that knowledge of effective coping strategies, as well as the ability to plan and regulate movements, plays a major role in the active condition but is less relevant in the passive condition. Individuals with cognitive impairment (indicative of early-stage dementia) may gradually lose such knowledge and planning abilities, potentially leading to impairments in goal-directed movement, including early forms of apraxia observed in neurodegenerative conditions such as Alzheimer's disease (31, 32). This may also affect anticipatory control and movement success (33), thereby reducing the capacity to manage potentially painful motor situations. Additionally, self-elicitation of pain requires awareness that one's own actions will produce discomfort, which may be diminished in dementia (34). In cognitively healthy individuals, however, self-elicited pain is typically perceived as less intense (35).

Overall, these remain plausible speculations that require further empirical evidence, particularly regarding whether actively movement-induced and self-elicited pain assessment protocols are more sensitive in detecting increased pain perception in cognitively impaired individuals. Accumulated evidence on the impact of dementia-related cognitive impairment on experimental pain responses is still mixed. Studies relying on non-verbal indicators, such as facial expressions (5, 20, 36, 37) and nociceptive flexion reflexes (28), predominantly suggest increased responsiveness to noxious stimulation. In contrast, measures such as pain detection and tolerance thresholds tend to indicate no change or even reduced sensitivity in individuals with dementia (38, 39).

A further aim of the study was to identify factors influencing pain during everyday motor tasks. As expected, participants reporting higher clinical pain during the geriatric pain interview (session 1), also reported greater pain during everyday motor tasks (session 2). Moreover, higher experimental pain sensitivity was associated with higher pain ratings during everyday motor tasks. Most interestingly, cognitive functioning also significantly contributed to the prediction of movement-related pain. Despite the very mild and limited range of cognitive impairment in our sample, cognitive functioning explained a comparable proportion of variance in pain responses to everyday motor tasks as clinical pain and experimental pain sensitivity. This finding is particularly surprising, as cognitive status was assessed using a broader dementia assessment battery. Consequently, neuropsychological functions that are especially relevant in this context, namely motor action planning and monitoring, as well as memory for successful motor maneuvers, were not specifically evaluated, which may represent an important objective for future studies.

### 4.2 Limitations

One limitation of the study is the restricted variability in cognitive status, as measured by CERAD, which allowed comparisons only between cognitively healthy individuals and those with cognitive impairments indicative of early-stage dementia. It therefore remains unclear whether the observed increase in pain during motor tasks would further intensify in individuals with more advanced cognitive impairment. A notable strength, however, is the advanced age of the sample, enhancing the study's relevance for geriatric pain research. However, our patients were not frail; frailty may still lead to different interactions between cognitive status, motor abilities, and pain experience. It should also be noted that the movement-related pain induced in this study was relatively mild, and the findings may not generalize to moderate or severe pain.

## 5 Conclusion

In summary, cognitively impaired individuals did not differ from cognitively healthy individuals in experimentally induced pain sensitivity but reported greater pain during everyday motor activities. This suggests that cognitive processes such as movement planning, knowledge of coping strategies, and behavioral control might influence pain perception in contexts requiring motor activities. Moreover, alongside clinical pain and experimental pain sensitivity, cognitive functioning emerged as an important factor in explaining movement-related pain. These findings highlight the importance of considering cognitive functioning when assessing pain in older adults and demonstrate the feasibility of studying pain during everyday motor tasks in individuals with early-stage dementia.

## Data Availability

The raw data supporting the conclusions of this article will be made available by the authors, without undue reservation.
